# Highly divergent isolates of chrysanthemum virus B and chrysanthemum virus R infecting chrysanthemum in Russia

**DOI:** 10.7717/peerj.12607

**Published:** 2022-01-05

**Authors:** Sergei N. Chirkov, Anna Sheveleva, Anastasiya Snezhkina, Anna Kudryavtseva, George Krasnov, Alexander Zakubanskiy, Irina Mitrofanova

**Affiliations:** 1 Department of Virology, Lomonosov Moscow State University, Moscow, Russia; 2 Kurchatov Genomic Center-NBG-NSC, Yalta, Russia; 3 Laboratory of Postgenomic Research, Engelhardt Institute of Molecular Biology, Russian Academy of Sciences, Moscow, Russia; 4 Department of Medical Genomics, Centre for Strategic Planning of FMBA of Russia, Moscow, Russia; 5 Plant Developmental Biology, Biotechnology and Biosafety Department, Nikita Botanical Gardens, Yalta, Russia

**Keywords:** Chrysanthemum virus R, Chrysanthemum virus B, RNA-Seq, Metatranscriptome, *Chrysanthemum morifolium* Ramat

## Abstract

**Background:**

Chrysanthemum is a popular ornamental and medicinal plant that suffers from many viruses and viroids. Among them, chrysanthemum virus B (CVB, genus *Carlavirus*, family *Betaflexiviridae*) is widespread in all chrysanthemum-growing regions. Another carlavirus, chrysanthemum virus R (CVR), has been recently discovered in China. Information about chrysanthemum viruses in Russia is very scarce. The objective of this work was to study the prevalence and genetic diversity of CVB and CVR in Russia.

**Methods:**

We surveyed the chrysanthemum (*Chrysanthemum morifolium* Ramat.) germplasm collection in the Nikita Botanical Gardens, Yalta, Russia. To detect CVB and CVR, we used RT-PCR with virus-specific primers. To reveal the complete genome sequences of CVB and CVR isolates, metatransciptomic analysis of the cultivars Ribonette, Fiji Yellow, and Golden Standard plants, naturally co-infected with CVB and CVR, was performed using Illumina high-throughput sequencing. The recombination detection tool (RDP4) was employed to search for recombination in assembled genomes.

**Results:**

A total of 90 plants of 23 local and introduced chrysanthemum cultivars were surveyed. From these, 58 and 43% plants tested positive for CVB and CVR, respectively. RNA-Seq analysis confirmed the presence of CVB and CVR, and revealed tomato aspermy virus in each of the three transcriptomes. Six near complete genomes of CVB and CVR were assembled from the RNA-Seq reads. The CVR isolate X21 from the cultivar Golden Standard was 92% identical to the Chinese isolate BJ. In contrast, genomes of the CVR isolates X6 and X13 (from the cultivars Ribonette and Fiji Yellow, respectively), were only 76% to 77% identical to the X21 and BJ, and shared 95% identity to one another and appear to represent a divergent group of the CVR. Two distantly related CVB isolates, GS1 and GS2, were found in a plant of the cultivar Golden Standard. Their genomes shared from 82% to 87% identity to each other and the CVB genome from the cultivar Fiji Yellow (isolate FY), as well as to CVB isolates from Japan and China. A recombination event of 3,720 nucleotides long was predicted in the replicase gene of the FY genome. It was supported by seven algorithms implemented in RDP4 with statistically significant *P*-values. The inferred major parent was the Indian isolate Uttar Pradesh (AM765837), and minor parent was unknown.

**Conclusion:**

We found a wide distribution of CVB and CVR in the chrysanthemum germplasm collection of the Nikita Botanical Gardens, which is the largest in Russia. Six near complete genomes of CVR and CVB isolates from Russia were assembled and characterized for the first time. This is the first report of CVR in Russia and outside of China thus expanding the information on the geographical distribution of the virus. Highly divergent CVB and CVR isolates have been identified that contributes the better understanding the genetic diversity of these viruses.

## Introduction

Chrysanthemum (genus *Chrysanthemum* in the family Asteraceae) is one of the most popular ornamental crops that is distributed globally as a cut flower, potted flowering plant, or garden plant (Engelmann & Hamacher, 2008; Trolinger et al., 2018). Chrysanthemum is also an important medicinal plant with anti-inflammatory, antioxidant, antimicrobial, hepatoprotective, and some other activities (Cui et al., 2014; Shahrajabian et al., 2019; Youssef et al., 2020). Over 20 viruses and viroids were shown to infect chrysanthemum reducing the production and decorative value of the plants (Mitrofanova, Zakubanskiy & Mitrofanova, 2018; Yan et al., 2020; Kubota et al., 2021; Liu et al., 2021). From these, chrysanthemum virus B (CVB, genus *Carlavirus* in the family *Betaflexiviridae*) is widespread in all chrysanthemum-growing regions (Trolinger et al., 2018). CVB induces leaf mosaic, mottling, or vein clearing in some chrysanthemum cultivars; often infected plants develop no visible symptoms (Hollings, 1957). Another proposed member of the genus *Carlavirus*, chrysanthemum virus R (CVR), has been recently discovered in China in plants displaying severe stunting (Wang et al., 2018).

Carlaviruses are transmitted from plant to plant mechanically, by aphids in a non-persistent manner, and through vegetative propagation. Filamentous virions are 610–700 nm in length and 12–15 nm in diameter. Single-stranded positive-sense genomic RNA of carlaviruses of 8–9 kb is 5′-capped and polyadenylated at the 3′-end. The genome comprises six open reading frames (ORF), encoding the replicase (ORF1), the movement proteins (ORFs 2, 3, and 4), the coat protein (CP) (ORF5), and the cysteine-rich (nucleic acid-binding) protein (CRP) (ORF6). Carlavirus genome also includes 5′- and 3′-terminal untranslated regions (UTR) and two or three short intergenic regions (IGR) that separate ORF1 from ORF2, ORF4 from ORF5, and if present, ORF5 from ORF6. Replicase protein contains methyltransferase (MTR), 2OG-Fe (II) oxygenase (OXY), papain-like proteinase (P-Pro), helicase (HEL), and RNA-dependent RNA-polymerase (RdRp) domains. A highly variable region (HVR) was identified between MTR and OXY domains in the betaflexiviridae replicase protein. Overlapping ORF2, ORF3, and ORF4 are organized as triple gene block (TGB) and encode TGB1, TGB2, and TGB3 proteins. CRP has an arginine-rich nuclear localization signal (NLS) and zinc finger (ZF) motif located adjacent to NLS (Morozov & Solovyev, 2003; Adams et al., 2012; Fujita et al., 2018).

To date, viruses infecting chrysanthemum have practically not been studied in Russia. The chrysanthemum germplasm collection, which is maintained in the Nikita Botanical Gardens (NBG), Yalta, Russia, includes as many as 180 cultivars and hybrid forms and is the largest in Russia. Some plants exhibit mosaic, leaf distortion, chlorotic mottle, vein clearing, and banding suggesting virus infection. Using a limited number of chrysanthemum samples (twelve cultivars were surveyed), CVB and tomato aspermy cucumovirus from the family *Bromoviridae* (TAV) were recently detected by ELISA and RT-PCR on local and introduced cultivars (Zakubanskiy et al., 2021) prompting further investigation of chrysanthemum viruses in these plantings.

The objective of this work was to study the prevalence and genetic diversity of CVB and CVR in Russia. These viruses were found in some chrysanthemum cultivars during surveys of the NBG collection. CVR was first reported in Russia and outside China. Nearly complete genomes of several Russian CVB and CVR isolates were determined using the high-throughput sequencing approach (HTS). Highly divergent variants of these viruses were discovered and characterized.

## Materials and Methods

### Sampling

We gathered leaf samples in the chrysanthemum (*Chrysanthemum morifolium* Ramat.) germplasm collection of the Nikita Botanical Gardens (44.51N; 34.23E) from August to October 2019–2020. Plants displaying symptoms of supposed virus infections were initially selected. Samples of plants with unclear symptoms and symptomless plants were also taken. Three to four leaves collected from each plant were pooled for subsequent analyses. Each plant was analyzed separately.

### RNA isolation, RT-PCR, and Sanger sequencing

We tested leaf samples for CVR and CVB using RT-PCR. Total RNA was isolated using RNeasy Plant Mini Kit (Qiagen, Hilden, Germany). Random hexamer primers and MMLV reverse transcriptase (Evrogen, Moscow, Russia) were employed for the first-strand cDNA synthesis. To detect CVB, primers P1/P2 amplifying the entire CP gene were used for PCR according to the original protocol (Ram et al., 2005). CVR was detected using primers CV1zF/CV807R, which target the 5′-terminal sequence of the replicase gene (Wang et al., 2018). The primer CV1zF (5′-ATGGCGCTCACTTTCCGCAGCC-3′) is a modified version of the original forward primer CV1F (Wang et al., 2018) that was found to be fitted better for the successful detection of Russian CVR isolates. The HVR of the CVR replicase gene was amplified using the primers CV1406F/CV2353R as described previously (Wang et al., 2018). The 3′-terminal CVR genome region covering the entire TGB3 and CP genes was amplified using the forward primer CV7162F (Wang et al., 2018) and a newly designed reverse primer CVR-R (5′-TATGACCCCTAGCCTTTTGA-3′), which target ORF3 and ORF6 of the CVR genome, respectively. A number of primers, recognized different regions of the CVR replicase gene, were also designed to confirm the HTS results. Information about primers used in this work is summarized in Table S1. Amplification was performed using the proof-reading Encyclo DNA polymerase (Evrogen, Moscow, Russia) and included the following cycles: denaturation at 94 °C for 30 s, primer annealing at 56 °C for 30 s, and elongation at 72 °C for 1 to 2 min depending on the size of expected PCR product, for 35 cycles with a final extension at 72 °C for 10 min. Amplicons were analyzed by 1.5% agarose gel electrophoresis using ethidium bromide staining and MultiDoc-It Digital Imaging System (Analytik Jena GmbH, Jena, Germany). Appropriate PCR products were purified from agarose gel with BC022 Cleanup Standard Kit (Evrogen, Moscow, Russia) and were subjected to Sanger sequencing in both directions using Evrogen facilities. If several plants of the same cultivar were found to be infected, PCR products from only one of them were sequenced.

### Assembly of viral genome sequences from RNA-Seq data

We generated three DNA libraries using polyA-enriched fraction of total RNA extracted from plants of the cultivars Ribonette, Fiji Yellow, and Golden Standard with mixed CVB and CVR infection. This fraction was obtained using a K0600 full-size poly(A) mRNA purification magnetic beads kit (Sileks, Moscow, Russia) according to the manufacturer’s protocol. TruSeq Stranded mRNA Library Prep Kit (Illumina, San Diego, CA, USA) was employed for the сDNA libraries synthesis, according to the manufacturer’s instructions. The libraries were sequenced on MiSeq Illumina platform. Reads were trimmed and filtered using Trimmomatic v.0.39 (Bolger, Lohse & Usadel, 2014). Next, contigs were assembled *de novo* using Trinity 2.11 with default settings (Grabherr et al., 2011). The virus-specific contigs were identified by BLASTn search using GenBank full-length viral genome database (https://ftp.ncbi.nlm.nih.gov/genomes/genbank/viral/assembly_summary.txt).

### Analysis of assembled genomes

Multiple alignments of genomic sequences using ClustalW, determination of their nucleotide (nt) and amino acid (aa) identities as well as phylogenetic analysis using the neighbor-joining algorithm and Kimura 2-parametric model were performed by the MEGA7 program (Kumar, Stecher & Tamura, 2016). Sequences of all available CVR and CVB isolates were retrieved from GenBank and employed for the analyses. Recombination detection program RDP4.46 (Martin et al., 2015) was used to search for recombination in CVR and CVB genomes using default settings with the exception that ‘sequences are linear’ and ‘list events detected by > 6 methods’ options were chosen.

## Results

### Prevalence of CVB and CVR in chrysanthemum collection

A total of 90 chrysanthemum plants of 22 most popular cultivars and one hybrid were surveyed (Table 1). By RT-PCR, CVB was detected in plants of 16 cultivars and hybrid 7–15. CVR was identified in eight cultivars and hybrid 7–15. PCR products of the expected sizes 948 base pairs (bp) and 747-bp were amplified in the samples infected with CVB and CVR, respectively (Fig. S1). A total of 52 plants (58%) were infected with CVB and 39 plants (43%) with CVR. The data presented in Table 1 show that CVB and CVR are mostly distributed across the collection independently to one another, although mixed infection was detected in 21 plants (23%). These viruses were detected in plants exhibiting mild mosaic, mottle or pale green spots and in symptomless plants as well suggesting that such the symptoms were non-specific for CVR and CVB (Fig. S2). In addition, these viruses were not detected in plants of the cultivar Yunost with symptoms of mottle on the leaves. No stunting was observed among tested plants. It is possible that the symptoms manifested in a cultivar-dependent manner. Neither CVB nor CVR were detected in the cultivars Dayon, Milka Pink, Plamya, and Yunost. Thus, we detected CVR in Russia and outside China for the first time expanding the information on the geographic distribution of the virus. We also demonstrated that CVR can be widespread on chrysanthemum.

**Table 1:** Incidence of chrysanthemum virus B (CVB) and chrysanthemum virus R (CVR) in chrysanthemum cultivars studied in this work.

Cultivar	Сultivar origin	Virus detected^1^					
		CVB			CVR		
		Plants tested/infected	Genome region sequenced^2^	GenBank accession number	Plants tested/infected	Genome region sequenced^2^	GenBank accession number
Antonov	Introduced	5/2	CP	MH678698	5/0	–	–
Chita	Introduced	5/5	CP	MH678699	5/3	Replicase; TGB3-CP	MZ340427 MZ340434 MZ231088
Dalistar Yellow	Introduced	3/3	CP	MH678700	3/0	–	–
Dayon	Introduced	4/0	–	–	4/0	–	–
Demurral Lilac	Introduced	6/2	CP	MH678701	6/0	–	–
Diana	Introduced	4/1	CP	MH678702	4/0	–	–
Fiji Yellow	Introduced	1/1	Near complete genome	MZ514910	1/1	Near complete genome	MZ514907
Golden Standard	Introduced	3/3	Near complete genome	MZ514908 MZ514909	3/1	Near complete genome	MZ514905
Grazia	Local	3/1	CP	MZ231085	3/0	–	–
Hybrid 7–15	Local	7/7	CP	MH678697	7/2	Replicase; TGB3-CP	MZ340424 MZ340430 MZ231086
Ludmila	Local	2/2	CP	MZ231083	2/0	–	–
Milka Pink	Introduced	3/0	–	–	3/0	–	–
Plamya	Local	4/0	–	–	4/0	–	–
Perfection Red	Introduced	1/1	CP	MZ231081	1/0	–	–
Ribonette	Introduced	5/4	Partial genome	MZ514911	5/1	Near complete genome	MZ514906
Sheer Purple	Introduced	1/1	CP	MZ231082	1/0	–	–
Simfonia	Local	3/3	CP	MH678703	3/0	–	–
Sirenevye Dali	Local	6/0	–	–	6/1	Replicase; TGB3-CP	MZ340425 MZ340433 MZ231090
Skazka	Local	1/0	–	–	1/1	Replicase; TGB3-CP	MZ340428 MZ340435 MZ231089
Snowdown White	Introduced	3/3	CP	MZ231084	3/0	–	–
Yuzhnaya Notch	Local	2/0	–	–	2/1	Replicase; TGB3-CP	MZ340429 MZ340432 MZ231087
Yunost	Local	5/0	–	–	5/0	–	–
Zolotovoloska	Introduced	13/13	CP	MH678704	13/13	Replicase; TGB3-CP	MZ340426 MZ340431 MZ340436

**Notes:**

1 RT-PCR.

2 TGB3: triple gene block, gene 3; CP: coat protein gene; Replicase: 5′-terminal region and hypervariable region of the replicase gene; Partial genome: (Cter) replicase-TGB1-TGB2-TGB3-CP-CRP-3′-UTR.

“-” - no data.

### Analysis of partial CVR genome sequences

Sequencing of 747-bp PCR products, generated in CVR-positive samples, showed that CVR isolates from the cultivars Sirenevye Dali, Skazka, Zolotovoloska, Golden Standard, Chita, Yuzhnaya Notch, and hybrid 7–15 were, in average, 96% identical to each other and the corresponding genomic region of the Chinese isolate BJ (MG432107). Until recently, the latter one was the only CVR isolate, which a full-length genome was available. At the same time, isolates from the cultivars Ribonette and Fiji Yellow shared 95% identity to each other and were only 83% identical to the rest ones and the isolate BJ, suggesting that they may represent a genetically distant CVR group. To test this assumption, we attempted to sequence two other genome regions of the Russian CVR isolates. The PCR products of the expected sizes 946 and 1,420-bp were generated by amplification of the hypervariable and 3′-terminal genomic regions, respectively, in most samples except the cultivars Fiji Yellow and Ribonette (Fig. S3). Partial genome sequences of CVR isolates from the cultivars Sirenevye Dali, Skazka, Zolotovoloska, Chita, Yuzhnaya Notch, and hybrid 7–15 were deposited in GenBank under accession numbers MZ231086 to MZ231090 and MZ340424 to MZ340436. We used these sequences for the phylogenetic analysis of three genomic regions. The HVR and TGB3-IGR2-CP sequences of the isolates from the cultivars Ribonette and Fiji Yellow were retrieved from their full-length genomes (see below). The trees were congruent and the isolates from the Ribonette and Fiji Yellow cultivars always formed a separate clade with 100% bootstrap values further supporting the idea that they belong to a divergent group of CVR (Fig. S4).

### Analysis of partial CVB genome sequences

**Figure 1:**
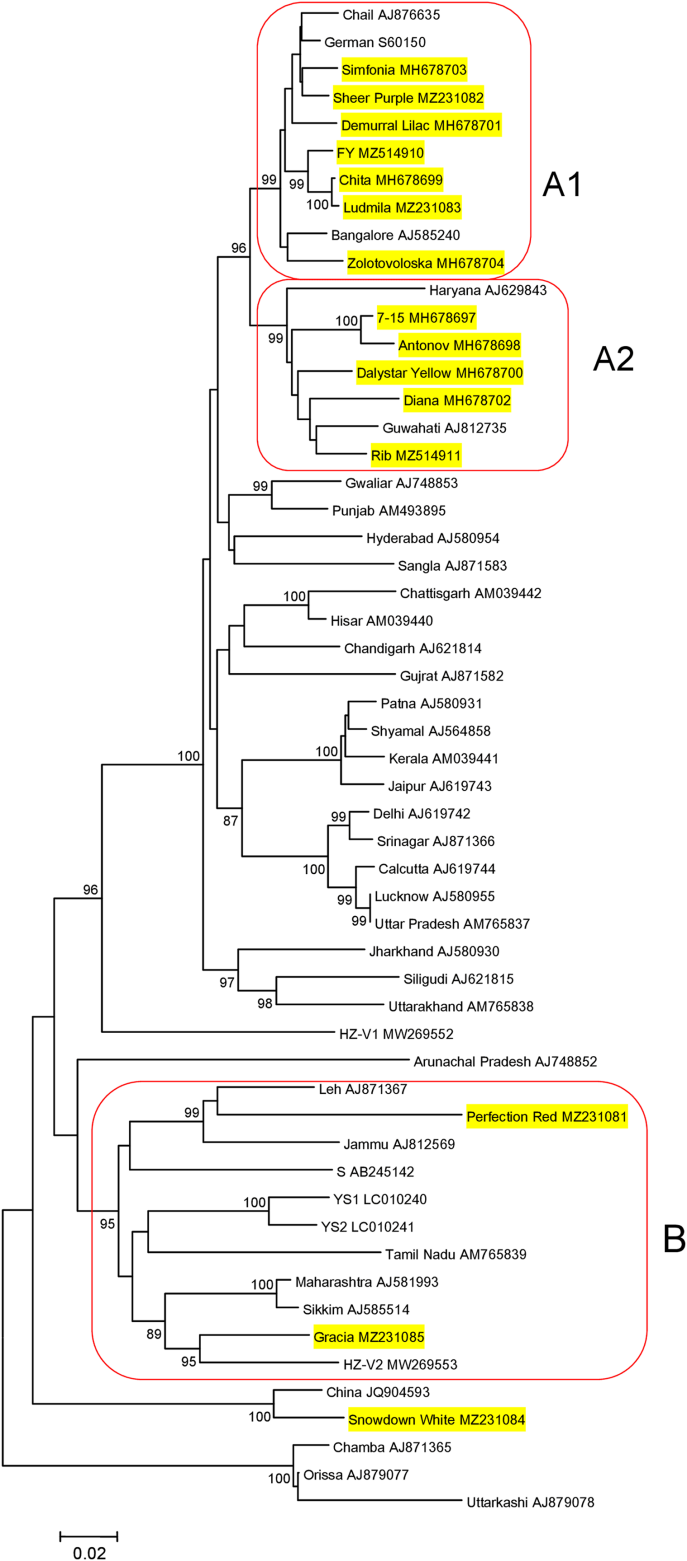
Phylogenetic analysis of the chrysanthemum virus B coat protein gene. The tree was reconstructed using the neighbour-joining algorithm implemented in MEGA7 (Kumar, Stecher & Tamura, 2016). Names of isolates and their GenBank accession numbers are shown at the end of branches. Bootstrap values (>85%) from 1,000 replicates are displayed next to the corresponding nodes. Russian isolates are in yellow. Clusters A1, A2, and B are highlighted. The scale bar indicates the number of substitutions per nucleotide.

Sequences of 948-bp PCR products, generated in CVB-positive samples and encompassed the entire CP gene, were deposited in GenBank under accession numbers MZ231081–MZ231085. Their phylogenetic analysis together with the CP of isolates from Russia (MH678697−MH697704) (Zakubanskiy et al., 2021), India, Korea, China and Japan available in GenBank showed that most Russian isolates were distributed among three groups (A1, A2, and B) as supported by high bootstrap values (Fig. 1). Within a group, the nt identities of the Russian isolates ranged from 95.6% to 99.7% in group A1 and 93.5% to 98.5% in group A2. The CVB isolates from the cultivars Perfection Red and Gracia (group B) were 82.5% identical to one another and 77.5% to 83.6% to the isolates from groups A1 and A2, indicating higher CP variability in group B. The isolate from the cultivar Showdown White was out of these groups and was most closely related to a Chinese isolate (JQ904593), sharing 96% identity. We failed to sequence the CP gene of the isolate from the cultivar Golden Standard due to a large number of ambiguous positions at the corresponding chromatograms suggesting that this cultivar can be co-infected with divergent CVB isolates (Data S1).

### Metatranscriptomic sequencing of samples from the cultivars Ribonette, Fiji Yellow, and Golden Standard

Partial genome sequences analysis showed that CVR isolates from the cultivars Ribonette and Fiji Yellow (named X6 and X13, respectively) appear to represent a divergent group of the virus, while that from the cultivar Golden Standard (isolate X21) is closer to other Russian isolates and the Chinese isolate BJ. On the other hand, the CVB isolates from the cultivars Ribonette and Fiji Yellow (named Rib and FY, respectively) are assigned to different phylogenetic groups considering the CP sequences. The cultivar Golden Standard is probably infected with divergent CVB isolates. Based on partial genome sequences, the samples from the cultivars Fiji Yellow, Golden Standard, and Ribonette were selected to determine CVR and CVB complete genomes using HTS.

A total of 12.4, 13.7, and 25.6 million of pair-end 150 nt reads were generated in the samples from the cultivars Ribonette, Fiji Yellow, and Golden Standard, respectively. CVB-, CVR-, and TAV-related reads were identified in each of these three samples. The proportion of virus-related reads is presented in Table 2. The raw reads were deposited in the NCBI Sequence Read Archive (SRA) (https://www.ncbi.nlm.nih.gov/bioproject/PRJNA751454/). No reads related to other viruses were detected by RNA-Seq analysis. Further we focused on the characterization of two carlaviruses genomes.

**Table 2:** The proportion of virus-related reads mapped to assembled contigs, %.

Virus	Proportion of virus-related reads in the sample:		
	Ribonette	Fiji yellow	Golden standard
CVB	17.8	9.0	12.9
CVR	8.5	10.0	18.4
TAV	0.8	0.01	7.9

**Notes:**

CVR, chrysanthemum virus R.

CVB, chrysanthemum virus B.

TAV, tomato aspermy virus.

The assembled CVR-related contigs covered the whole genome of the reference isolate BJ (MG432107, 8,990 nt) except 4 to 12 nt at the 5′-end of the genome. Five genome regions of the total length 4,046 nt (about of half of the genome) were re-sequenced by the Sanger method using primers presented in Table S1. The sequences determined by HTS and the Sanger method were shown to be identical. The near complete genomes of CVR isolates from the cultivars Ribonette (isolate X6; 8,878 nt), Fiji Yellow (X13; 8,872 nt), and Golden Standard (X21; 8,873 nt) were deposited in GenBank under accession numbers MZ514905 to MZ514907.

The assembled CVB-related contigs from the cultivars Golden Standard and Fiji Yellow covered the whole genome of the reference isolate S (AB245142, 8,990 nt) except 12 to 19 nt at the 5′-end of the genome. Two different CVB genomes (designated GS1 and GS2) were assembled in the cultivar Golden Standard. The near complete genomes of the CVB isolates GS1 (8,977 nt), GS2 (8,976 nt), and FY (8,970 nt) were deposited in GenBank under accession numbers MZ514908 to MZ514910. The CVB genome from the cultivar Ribonette (isolate Rib) was failed to assemble completely. The partial genomic sequence of 5,174 nt covering the C-terminal part of the replicase gene, TGB, CP, and CRP genes as well as 3′-UTR was deposited in GenBank under accession number MZ514911. The CP sequences of the isolates FY and Rib determined by the HTS and the Sanger method were identical.

### Characterization of new CVR genomes

**Figure 2:**
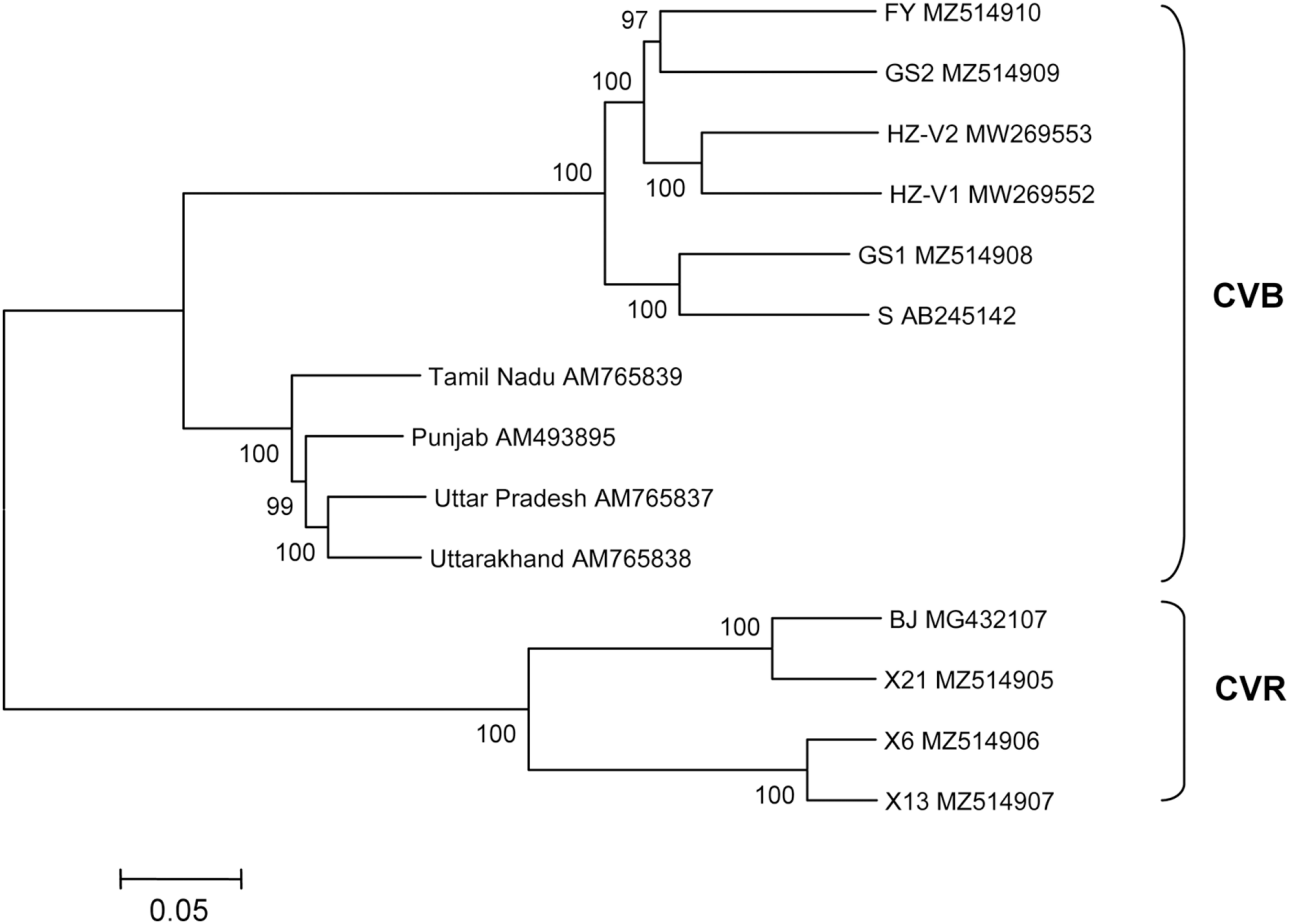
Phylogenetic analysis of complete genomes of chrysanthemum virus B (CVB) and chrysanthemum virus R (CVR). The tree was reconstructed using neighbour-joining algorithm and Kimura 2-parametric model implemented in MEGA7 (Kumar, Stecher & Tamura, 2016). Names of isolates and their GenBank accession numbers are shown at the end of branches. Bootstrap values from 1,000 replicates are displayed next to the corresponding nodes. The scale bar indicates the number of substitutions per nucleotide.

**Figure 3:**
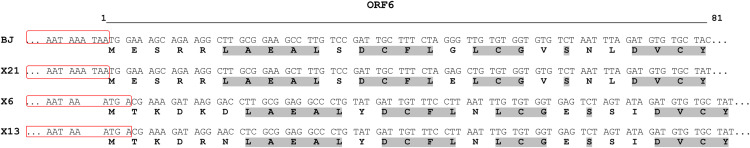
Alignment of N-terminal part of the ORF6-encoded cysteine-rich protein of chrysanthemum virus R isolates. The names of the isolates are given in the left column. For each isolate, the upper and lower rows show nucleotide and predicted amino acid sequences, respectively. The 3′-ends of ORF5 are boxed. Conserved amino acids are in light grey. The line above the alignment covers the 5′-ends of ORF6 from nucleotides 1 to 81.

The structure of newly sequenced CVR genomes was typical of carlaviruses. These comprise six ORFs, 5′- and 3′-UTRs, and two IGRs between ORF1 and ORF2 as well as between ORF4 and ORF5. Complete genomes of the Russian isolates and Chinese isolate BJ were compared (Table 3). Isolate X21 was 92% identical to the BJ and similar to the latter in the size and organization of all genomic elements. In contrast, genomes of the isolates X6 and X13 were 76% to 77% identical to the X21 and BJ and shared 95% identity to one another. ORF4 was the most variable genome region both at the nt and aa levels. In opposite, ORF5 and ORF6 were the most conserved. Phylogenetic analysis showed that isolates BJ, X21 and X6, X13 formed two separate clades with 100% bootstrap support (Fig. 2). Using NCBI Conserved Domain Search Service (https://www.ncbi.nlm.nih.gov/Structure/cdd/wrpsb.cgi), the MTR, OXY, P-Pro, HEL and RdRp domains were identified in the X6, X13, and X21 replicase proteins at identical or close positions. At the same time, multiple alignment of ORF1s revealed a number of indels that resulted in several aa deletions located between MTR, OXY, and P-Pro domains. Another aa deletion was predicted in HEL domain of the X6 and X13 (Fig. S5). These indels were confirmed by Sanger sequencing of the corresponding genome regions using appropriate primers (Table S1). Due to these distinctions, X6 and X13 ORF1s are shorter by 6 nt and the encoded replicase protein is shorter by 2 aa when comparing with the isolates X21 and BJ. Furthermore, IGR1s of the X6 and X13 are of 27 nt length that shorter by 1 nt than those in the isolates X21 and BJ. Also, ORF3 and ORF5 in the isolates X21 and BJ are terminated with TAA and OFR6 with TGA. On the contrary, in the X6 and X13 the stop codon in ORF3 and ORF5 is TGA, whereas ORF6 is terminated with TAA. Because ORF5 and ORF6 overlap, replacing the TAA terminating codon with TGA in ORF5 of the X6 and X13 shifts putative ORF6 start AUG codon by 3 nt upstream. This shift resulted in the CRP elongation by 1 aa comparing to the X21 and BJ and in changes of aa sequence in the N-terminal region of the protein (Fig. 3). However, neither NLS (^48^RRRR^51^) nor ZF motifs (^58^CX_2_CX_12_CX_4_C^79^) were affected in the isolates X6 and X13.

**Table 3:** Nucleotide (nt) and amino acid (aa) identities (%) of open reading frames (ORF) and near complete genomes of Russian chrysanthemum virus R isolates vs Сhinese isolate BJ (MG432107).

Cultivar	Isolate	ORF1		ORF2		ORF3		ORF4		ORF5		ORF6		Whole genome
		nt	aa	nt	aa	nt	aa	nt	aa	nt	aa	nt	aa	
Golden Standard	X21	91	95	93	96	92	97	92	92	94	98	95	96	92
Ribonette	X6	75	85	80	87	74	86	73	70	80	93	81	83	77
Fiji Yellow	X13	75	84	80	86	75	85	72	70	79	92	82	84	76

Thus, based on near complete genome sequences and phylogenetic analysis, at least two divergent groups of CVR exist, which can be probably considered different strains of the virus.

In addition, the X6 and X13 genome analysis showed that these isolates were obviously not recognized by primers targeting HVR and 3′-terminal genome regions due to a large number of mismatches between the primers and the corresponding genome sequences (3 to 9 per a primer). In contrast, RT-PCR with primers CV1zF/CV807R, specific to the beginning of the polymerase gene, recognized even highly divergent isolates of the virus and apparently can be successfully applied for the CVR detection.

### Characterization of new CVB genomes

We assembled near complete genome sequences of the CVB isolates FY, GS1, and GS2. Typically for carlaviruses, six ORFs, 5′- and 3′-UTRs, and three IGRs between ORF1 and ORF2, ORF4 and ORF5, ORF5 and ORF6 were identified in the new genomes.

Seven complete CVB genomes were available in GenBank until recently. Four of these are from India (AM493895, AM765837−AM765839) (Singh et al., 2012), two were found in China on a new host *Gynura japonica* (MW269552–MW269553) (Lai et al., 2021), and another isolate S is from Japan (AB245142) (Ohkawa et al., 2007). The multiple alignments showed that the isolates GS1 and GS2 were 82% identical to each other and shared 82% to 84% identity with the FY. Overall, the Russian CVBs were 83% to 87% identical to the Chinese and Japanese isolates and 68% to 72% identical to the Indian ones. The Japanese isolate S was used as a reference to compare the Russian complete genomes (Table 4). ORF1 is the most variably part of the FY and GS2 genomes while ORF6 of the FY and GS1 is the most conserved. At the same time, ORF4 of the FY and ORF6 of the GS2 were only distantly related to their counterparts from other isolates. Phylogenetic analysis of the complete or near complete genomes of all available CVB isolates showed that the Indian isolates form a separate cluster (Fig. 2). Another cluster is represented by isolates from Russia, Japan, and China. The FY and GS2 form a separate clade, and the isolate GS1 is clustered with the Japanese isolate S.

**Table 4:** Nucleotide (nt) and amino acid (aa) identities (%) of open reading frames (ORF) and near complete genomes of Russian chrysanthemum virus B isolates vs Japanese isolate S (AB245142).

Cultivar	Isolate	ORF1		ORF2		ORF3		ORF4		ORF5		ORF6		Whole genome
		nt	aa	nt	aa	nt	aa	nt	aa	nt	aa	nt	aa	
Golden standard	GS1	86	92	87	93	85	95	86	92	87	94	95	97	87
	GS2	80	87	85	91	84	95	84	86	83	94	86	88	81
Fiji yellow	FY	80	86	82	88	86	93	77	86	83	93	90	96	80

In addition, the detection of two different CVB isolates, GS1 and GS2, in the same plant of the cultivar Golden Standard by HTS enables to explain why we failed to obtain a consensus sequence of the CP gene by Sanger sequencing. A large number of ambiguous positions at chromatograms is probably because the CPs of the GS1 and GS2 are identical only 83% at the nt level.

**Figure 4:**
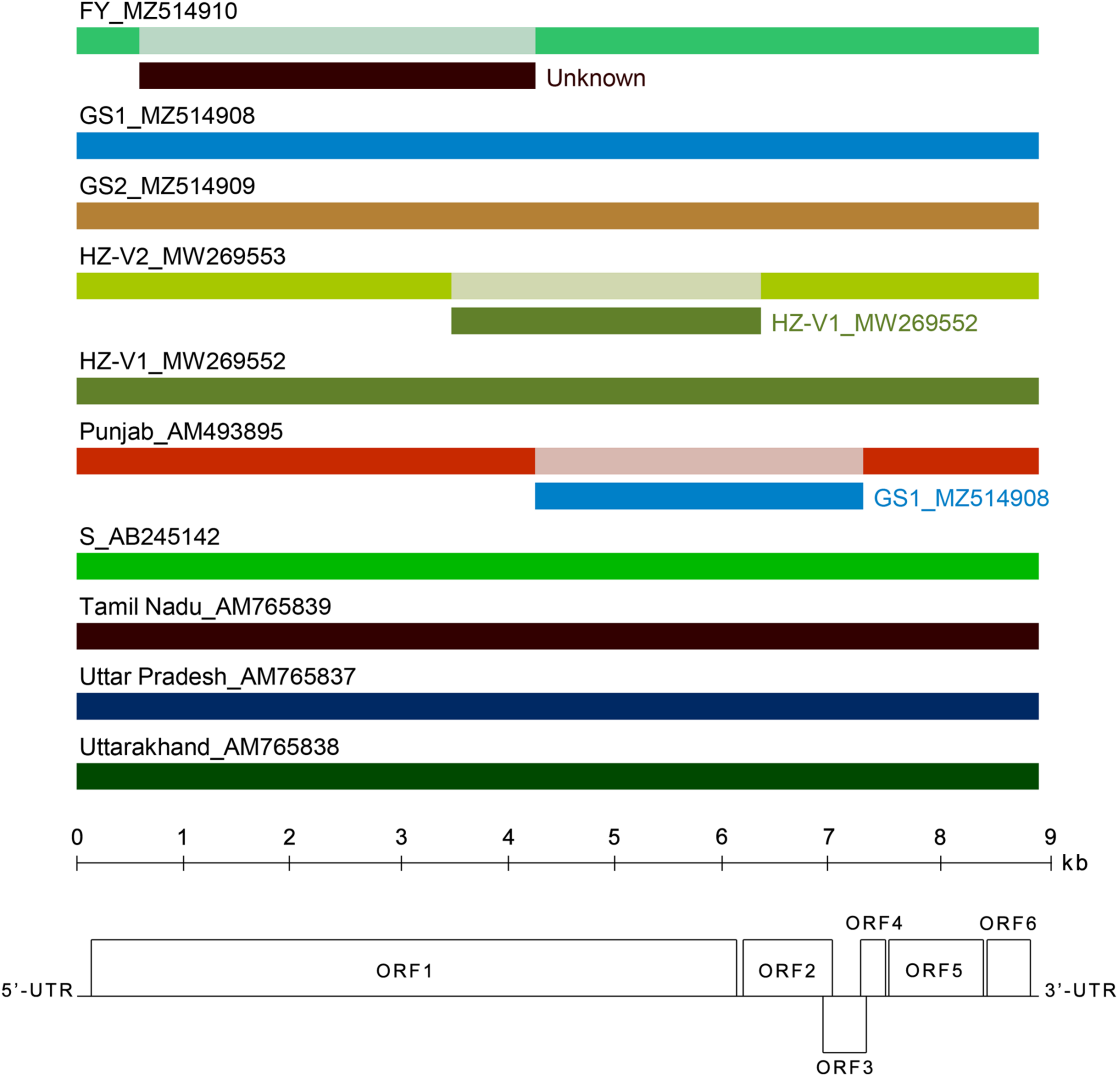
Analysis of recombination events in the alignment of ten full-length genomes of chrysanthemum virus B (CVB) isolates by the Recombinant Detection Program (RDP) v.4.46 (Martin et al., 2015). Summarized results of the recombination events detection are presented. The names of isolates and their GenBank accession numbers are indicated above the long bars. The names of inferred minor parents are indicated to the right of short bars beneath the corresponding isolate bars. The map of the CVB genome and the ruler are presented in scale below the graph.

Several recombination events (RE) were detected in the genomes of Indian and Japanese isolates previously (Singh et al., 2012). In this work, we looked for recombination in the extended alignment that included near complete genomes of new Chinese and Russian CVB. Three REs were predicted in the replicase gene (Fig. 4). The RE1 in the FY genome was supported by seven independent algorithms implemented in RDP4 with statistically significant *P*-values (RDP = 3.4 × 10^−208^, GENECONV = 4.9 × 10^−169^, BOOTSCAN = 7.2 × 10^−190^, MAXIMUM CHI SQUARE = 1.5 × 10^−49^, CHIMAERA = 5.4 × 10^−49^, SISCAN = 7.1 × 10^−121^, 3SEQ = 10^−146^, and 2.9 × 10^−169^ in average). The RE1 was bounded with breakpoints at positions 602 and 4,322, encompassing C-terminal part of MTR domain, the entire OXY and P-Pro domains, and the N-terminal part of HEL domain. The recombinant sequence is 3,720 nt, which is about 40% of the whole genome. The inferred major parent was the Indian isolate Uttar Pradesh (AM765837), and the minor parent was unknown. However, the isolates FY and Uttar Pradesh have only 88% identity beyond the recombinant part of their genomes. In addition, a BLASTn search along GenBank nucleotide collection (https://blast.ncbi.nlm.nih.gov/Blast.cgi) showed that the recombinant sequence was most closely related to the corresponding genomic region of the CVB isolates HZ-V2 sharing 79% identity. These data suggest that some other CVB isolates seemed to be the parents of the recombinant isolate FY. Apparently, closer isolates have to be found yet or their full-length genomes are not available at the moment. The RE1 was also confirmed by phylogenetic analysis of CVB isolates (Fig. S6). The trees reconstructed from different genome regions corresponding to the putative recombinant sequence of the isolate FY and to the rest of the genome were incongruent suggesting recombination (Chare & Holmes, 2006). The RE2 and RE3 were also identified in the isolates Punjab and HZ-V2 with a high degree of confidence (the average *P*-values were 3.8 × 10^−151^ and 1.2 × 10^−130^, respectively).

## Discussion

Plant viruses with RNA genomes have a great potential to generate the genetic diversity due to large population sizes, short generation time, and high rate of mutation because viral RNA polymerase lacks proofreading activity (Pagan, 2018). Metagenomics based on HTS potentially allows identifying all the viruses and their variants that infect a given plant and sequencing their genomes. Since the pioneering work of Adams et al. (2009), HTS has been widely used for the analysis of viruses of agricultural crops (Maliogka et al., 2018; Maree et al., 2018; Villamor et al., 2019). The main task of this work was to sequence and analyze the genomes of Russian CVB and CVR chrysanthemum isolates using HTS.

Three near complete genomes of CVR and three near complete genomes of CVB from Russia were sequenced and characterized for the first time. Seven complete or near complete genomes of CVB were deposited in GenBank until now. This is surprisingly small for a virus that infects an economically significant crop, has been known since 1957 (Hollings, 1957) and distributed worldwide. The only CVR isolate known to date has been discovered in China. Our study expands the information on the CVB and CVR geographical distribution and genetic diversity. Highly divergent isolates of these viruses have been identified. At least two divergent groups of CVR were revealed, which can be probably considered as different strains of the virus. The differences between these groups are mainly accumulated in ORF1, ORF4, and ORF6 encoding replicase, TGB3, and CRP, respectively.

In particular, we found that the CRP of CVR in the X6 and X13 isolates is 1 aa longer and differs in the N-terminal region from its counterparts of X21 and BJ isolates due to the shift of the ATG start codon (Fig. 3). Carlavirus CRP is known to be a pathogenicity determinant and to serve as an RNA silencing suppressor. Highly divergent N-terminal region determines symptoms patterns. So, CVB CRP induced leaf malformation while the potato virus M CRP induced whole plant stunting in *Nicotiana occidentalis* plants (Fujita et al., 2018). Since we observed neither malformation nor stunting among the chrysanthemum plants we studied, it is not clear whether the differences in the СRPs we have identified affect the manifestation of symptoms.

To the best of our knowledge, CVR was found only in China so far (Wang et al., 2018). In this work, the virus was detected in Russia and outside of China for the first time thus expanding the information on the geographical distribution of CVR. In particular, the virus was detected on cultivars introduced from Poland, Netherlands, and Ukraine many years ago. It cannot be ruled out that CVR may be distributed in Europe. The data presented in Table 1 also show that, in agreement with Wang et al. (2018), CVR seems to be widespread on chrysanthemum.

Recombination can play an important role in generating the CVB molecular diversity (Singh et al., 2007; Singh et al., 2012). Three REs were predicted in the CVB genomes in this work (Fig. 4). It should be noted however that the results we got from the recombination study were inconsistent with the previous data (Singh et al., 2012). In particular, the Japanese isolate S was shown to be a recombinant of Uttar Pradesh (AM765837) and Tamil Nadu (AM765839) isolates as major and minor parents, respectively. No recombination was detected in the isolate S in this work. Perhaps, these discrepancies are due to the different composition of the alignment that affects the RE detection. For example, the absence of one of the parents in the alignment can result in the misidentification of the recombinant genome (Martin et al., 2015). Whereas four Indian isolates and one Japanese isolate were studied by Singh et al. (2012), five more Russian and Chinese isolates were added in our work. On the other hand, REs in the FY and S genomes are very similar. Like the RE1, the recombination event in the isolate S is located in the replicase gene, bounded with two breakpoints at close positions 538 and 4,260, and is of the same length (3,722 nt). It is worthy to note that the FY isolate was found in a plant that was also infected with CVR making possible interspecific recombination between these two viruses. However, the inclusion of four full-length CVR genomes to the alignment revealed no additional REs in the CVB or CVR isolates (data not shown). Thus, recombination in CVB genomes needs further investigation by analyzing a larger number of isolates from different regions of the world.

Both CVB and CVR are members of the genus *Carlavirus*. High-throughput sequencing of the genomes of two related, albeit different viruses contained in the same sample can potentially result in the assembly of chimeric contigs. Nevertheless, all five partial genomic sequences of the CVR isolates X6, X13, and X21, obtained by the Sanger method, were identical to the corresponding genome regions determined by HTS. Furthermore, the indel-containing sequences of the replicase gene in CVR isolates (Fig. S4) were confirmed by Sanger sequencing. The CP sequences of the CVB isolates FY and Rib determined by HTS and Sanger methods were also identical. Near complete genomes of the Russian CVR isolates X6, X13, X21, and the CVB isolates FY, GS1, GS2 shared 52.0 to 52.9% identity that is typical for different species in the genus *Carlavirus* (Adams et al., 2012). Two different CVB isolates, GS1 and GS2, were identified in a plant of the cultivar Golden Standard. The corresponding assembled contigs had 82% identity to each other. The substitutions and short indels (distinguishing between these GS1 and GS2) were uniformly distributed along the whole contig length, which excludes the possibility that these contigs are technical artifacts (chimeras). Moreover, none of the RDP4 algorithms found evidence of recombination between these contigs. The average coverage of the isolate GS2 was five times higher than that for the isolate GS1.

In conclusion, we found a wide distribution of CVB and CVR in the chrysanthemum germplasm collection of the NBG. Six near complete genomes of CVR and CVB isolates from Russia were sequenced and characterized for the first time. Highly divergent isolates of CVB and CVR have been identified that contribute to the better understanding the genetic diversity of these viruses. This is the first report of CVR in Russia and outside of China thus expanding the information on the geographical distribution of the virus.

## Supplemental Information

10.7717/peerj.12607/supp-1 Supplemental Information 1 Primers used in this work.

10.7717/peerj.12607/supp-2 Supplemental Information 2 Representative analysis of RT-PCR products from chrysanthemum virus R (CVR)- and chrysanthemum virus B (CVB)-infected plants by agarose gel electrophoresis. The names of chrysanthemum cultivars are indicated above the picture. M-GeneRuler 100 bp DNA ladder Plus (Thermo Scientific). The arrows to the right of the picture indicate the CVR- and CVB-specific PCR products of 747 and 948 base pairs, respectively.

10.7717/peerj.12607/supp-3 Supplemental Information 3 Typical symptoms on the leaves of chrysanthemum cultivars Fiji Yellow (a), Ribonette (b), Golden Standard (c), Chita (d), Yunost (e), and hybrid 7–15 (f). **a–d** and **f** are co-infected with chrysanthemum virus R (CVR) and chrysanthemum virus B (CVB); **e**-no CVR and CVB was detected.

10.7717/peerj.12607/supp-4 Supplemental Information 4 Representative analysis of RT-PCR products from chrysanthemum virus R (CVR)-infected plants by agarose gel electrophoresis. The names of chrysanthemum cultivars are indicated above the picture. M-GeneRuler 100 bp DNA ladder Plus (Thermo Scientific). The arrows to the right of the picture indicates the CVR-specific PCR products of 747, 946, and 1,420 base pairs.

10.7717/peerj.12607/supp-5 Supplemental Information 5 Phylogenetic analysis of three genomic regions of chrysanthemum virus R isolates. A–5’-terminal region of the replicase gene; B–hypervariable region of the replicase gene; C–triple gene block 3 protein gene, intergenic region 2, and coat protein gene. The trees were reconstructed using the neighbour-joining algorithm and Kimura 2-parametric model implemented in MEGA7 (Kumar, Stecher & Tamura, 2016). Names of isolates and their GenBank accession numbers are shown at the end of branches. SK, YN, ZV, SD, Chita, X21, X6, and X13 are isolates from the cultivars Skazka, Yuzhnaya Notch, Zolotovoloska, Sirenevye Dali, Chita, Golden Standard, Ribonette, and Fiji Yellow, respectively. BJ is the Chinese isolate BJ and 7–15 is the Russian isolate from hybrid 7–15. Bootstrap values (>75%) from 1,000 replicates are displayed next to the corresponding nodes. The scale bars indicate the number of substitutions per nucleotide.

10.7717/peerj.12607/supp-6 Supplemental Information 6 Alignment of three regions of the chrysanthemum virus R (CVR) replicase protein. Alignment was employed using ClustalW implemented in MEGA7 program. Names of isolates are presented in the left column. Numerals above the alignment indicate amino acid positions (numbering according to the CVR Chinese isolate BJ replicase, MG432107). Deletions are in blue. C-terminal fragment of 2OG-Fe(II) Oxy domain and N-terminal fragment of helicase domain are in yellow and green, respectively.

10.7717/peerj.12607/supp-7 Supplemental Information 7 Phylogenetic analysis of chrysanthemum virus B genome regions corresponding to the recombinant sequence of the isolate FY (A) and to the rest of the genome (B). The Russian isolates are in yellow. The trees were reconstructed using the neighbour-joining algorithm implemented in MEGA7 (Kumar, Stecher & Tamura, 2016). Names of isolates and their GenBank accession numbers are shown at the end of branches. Bootstrap values from 1000 replicates are displayed next to the nodes. The scale bars indicate the number of substitutions per nucleotide.

10.7717/peerj.12607/supp-8 Supplemental Information 8 Golden Standard CVB chromatograms.

10.7717/peerj.12607/supp-9 Supplemental Information 9 Sequences deposited in GenBank.

## Additional Information and Declarations

### Data Availability

The following information was supplied regarding data availability: The raw sequence data are available at SRA in NCBI: PRJNA751454. The partial and complete genome sequences are available at GenBank: MZ231081–MZ231090, MZ340424–MZ340436, MZ514905–MZ514911 and available in the Supplemental File. The Golden Standard CVB chromatograms are available in the Supplemental File.
