# Supplementary material for: Highly divergent isolates of chrysanthemum virus B and chrysanthemum virus R infecting chrysanthemum in Russia
Source: PeerJ. 2022 Jan 5;10:e12607. doi: 10.7717/peerj.12607 (PMC8742542; doi:10.7717/peerj.12607)
Supplement: Supplemental Information 1 [file peerj-10-12607-s001.doc]

Table S1. Primers used in this work.

| **Virus** | **Primer name** | **Primer sequence (5'-3')** | **Genome positions^1^** | **Target gene** | **Amplicon size, base pairs** | **Reference** |
| --- | --- | --- | --- | --- | --- | --- |
| Chrysanthemum virus R (CVR) | CV1zF | ATGGCGCTCACTTTCCGCAGCC | 71 - 92 | Replicase | 747 | This work |
|  | CV807R | CAGTGGCTGCTCATAACCTTC | 797 - 817 |  |  | *Wang et al.,* 2018 |
|  | CV1406F | TGGCTGATGAACTGCTGGAT | 1407 - 1426 | Replicase | 946 | *Wang et al.,* 2018 |
|  | CV2353R | GGAACCGCCATTGTAAGTGTA | 2333 - 2353 |  |  | *Wang et al.,* 2018 |
|  | CVR-F1 | ATCGGGCTCCAGGCCTACAT | 1730 - 1749 | Replicase | 487 | This work |
|  | CVR-R1 | TGACCACGTGCTTCTCCTCT | 2191 - 2210 |  |  |  |
|  | CVR-F1 | ATCGGGCTCCAGGCCTACAT | 1730 - 1749 | Replicase | 510 | This work |
|  | CVR-R2 | CAGTGCTATGTCACAGGTGCA | 2213 - 2233 |  |  |  |
|  | CVR-F2 | AATATTGAAGGGCATGCAAC | 2522 - 2541 | Replicase | 446 | This work |
|  | CVR-R3 | CCATTAAAGTGCCTGGTGAA | 2939 - 2958 |  |  |  |
|  | CVR-F2 | AATATTGAAGGGCATGCAAC | 2522 - 2541 | Replicase | 446 | This work |
|  | CVR-R4 | CCATTAAAGTGTCTCGTGAA | 2939 - 2958 |  |  |  |
|  | CV7162F | ATCTACTACGGACGACCGAATA | 7162 - 7183 | Triple gene block protein 3 | 1420 | *Wang et al.,* 2018 |
|  | CVR-R | TATGACCCCTAGCCTTTTGA | 8562 - 8581 | Cysteine-rich protein |  | This work |
| Chrysanthemum virus B (CVB) | P1 | ATGCCTCCCAAACCGGCACCAGGTGAT | 7619 - 7645 | Coat protein | 948 | *Ram et al.*, 2005 |
|  | P2 | TTTATAATGTCTTATTATTCGCAT | 8544 - 8568 |  |  |  |

^1^Numbering according to CVR isolate BJ (MG432107) genome and CVB isolate S (AB245142) genome.
