# Supplementary material for: Highly divergent isolates of chrysanthemum virus B and chrysanthemum virus R infecting chrysanthemum in Russia
Source: PeerJ. 2022 Jan 5;10:e12607. doi: 10.7717/peerj.12607 (PMC8742542; doi:10.7717/peerj.12607)
Supplement: Supplemental Information 2 — The names of chrysanthemum cultivars are indicated above the picture. M-GeneRuler 100 bp DNA ladder Plus (Thermo Scientific). The arrows to the right of the picture indicate the CVR- and CVB-specific PCR products of 747 and 948 base pairs, respectively. [file peerj-10-12607-s002.pdf]

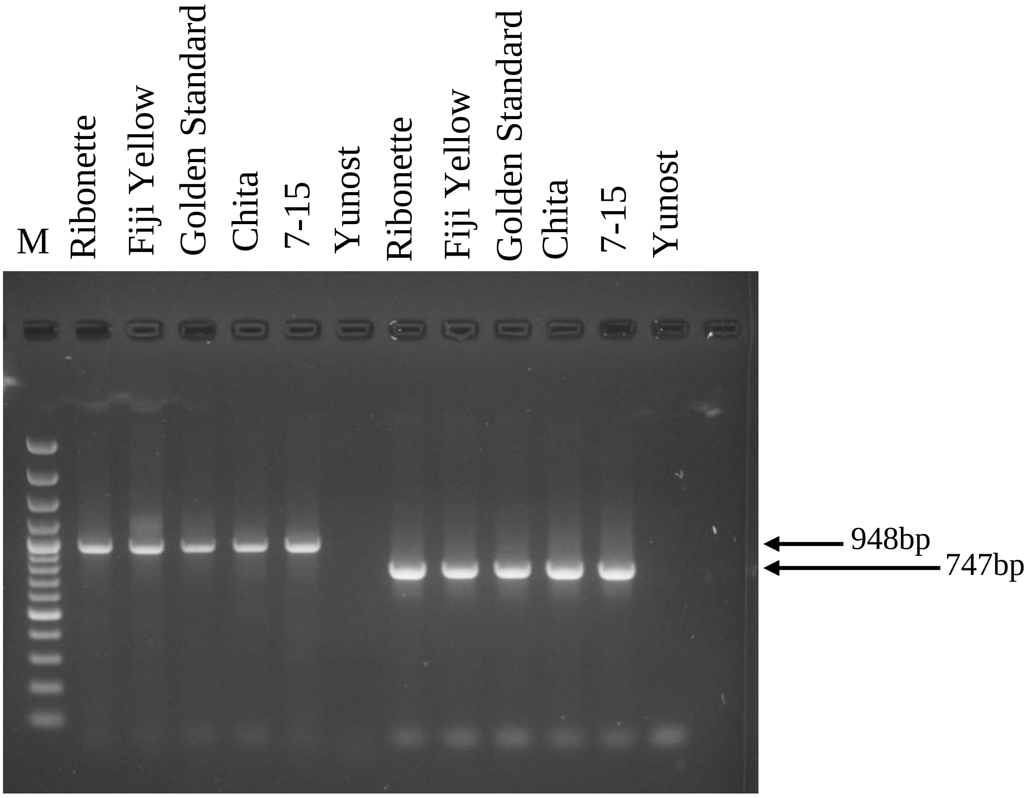

Figure S1. Representaive analysis of RT-PCR products from chrysanthemum virus R (CVR)- and chrysanthemum virus B (CVB)-infected plants by agarose gel electrophoresis. The names of chrysanthemum cultivars are indicated above the picture. M - GeneRuler 100 bp DNA ladder Plus (Thermo Scientific). The arrows to the right of the picture indicate the CVR- and CVB-specific PCR products of 747 and 948 base pairs, respectively.
