# Supplementary material for: Highly divergent isolates of chrysanthemum virus B and chrysanthemum virus R infecting chrysanthemum in Russia
Source: PeerJ. 2022 Jan 5;10:e12607. doi: 10.7717/peerj.12607 (PMC8742542; doi:10.7717/peerj.12607)
Supplement: Supplemental Information 3 — a–d and f are co-infected with chrysanthemum virus R (CVR) and chrysanthemum virus B (CVB); e-no CVR and CVB was detected. [file peerj-10-12607-s003.pdf]

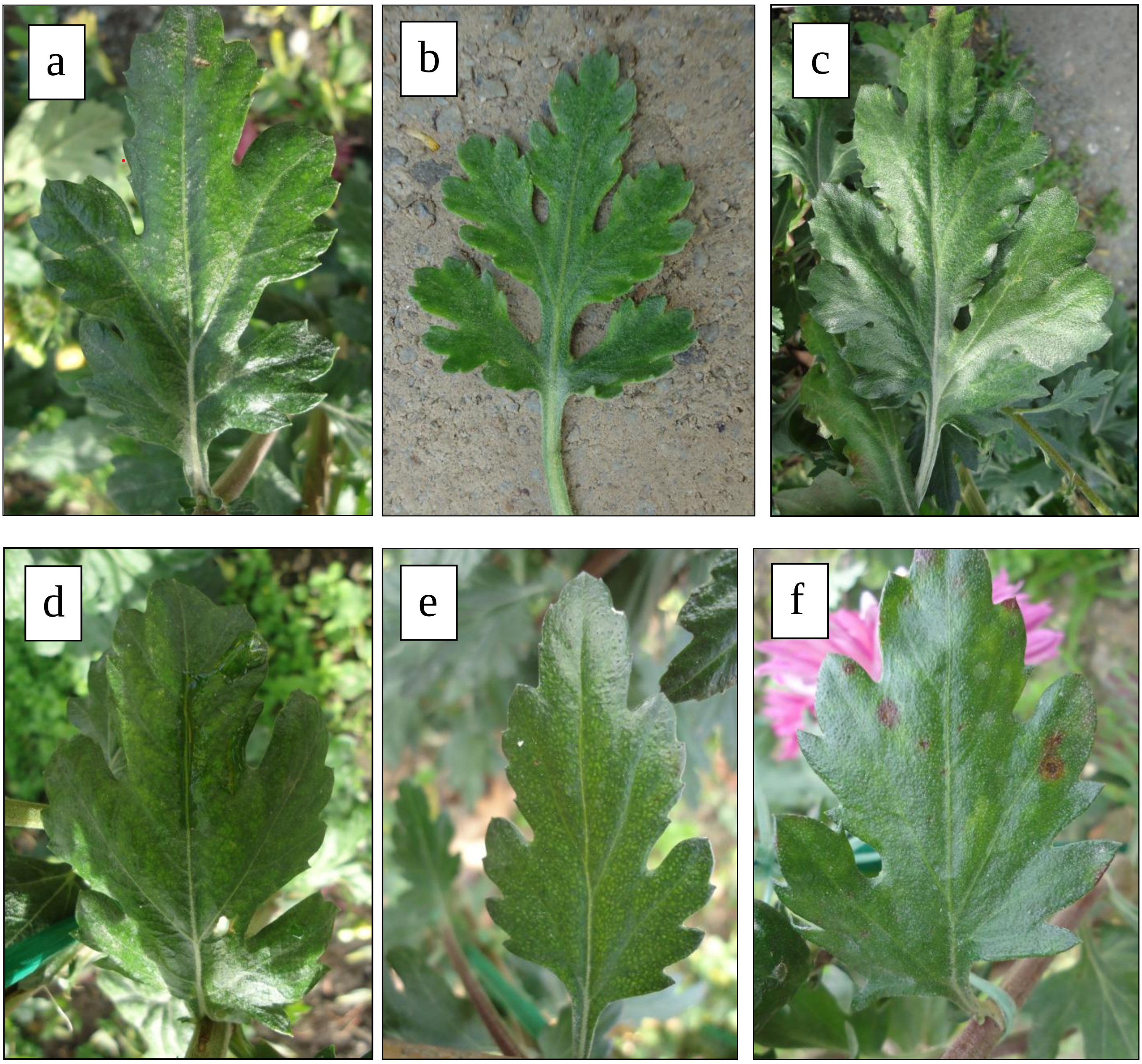

Figure S2. Typical symptoms on the leaves of chrysanthemum cultivars Fiji Yellow (**a**), Ribonette (**b**), Golden Standard (**c**), Chita (**d**), Yunost (**e**), and hybrid 7-15 (**f**). Samples **a - d** and **f** are co-infected with chrysanthemum virus R (CVR) and chrysanthemum virus B (CVB); **e** - no CVR and CVB were detected.
