# Supplementary material for: Highly divergent isolates of chrysanthemum virus B and chrysanthemum virus R infecting chrysanthemum in Russia
Source: PeerJ. 2022 Jan 5;10:e12607. doi: 10.7717/peerj.12607 (PMC8742542; doi:10.7717/peerj.12607)
Supplement: Supplemental Information 4 — The names of chrysanthemum cultivars are indicated above the picture. M-GeneRuler 100 bp DNA ladder Plus (Thermo Scientific). The arrows to the right of the picture indicates the CVR-specific PCR products of 747, 946, and 1,420 base pairs. [file peerj-10-12607-s004.pdf]

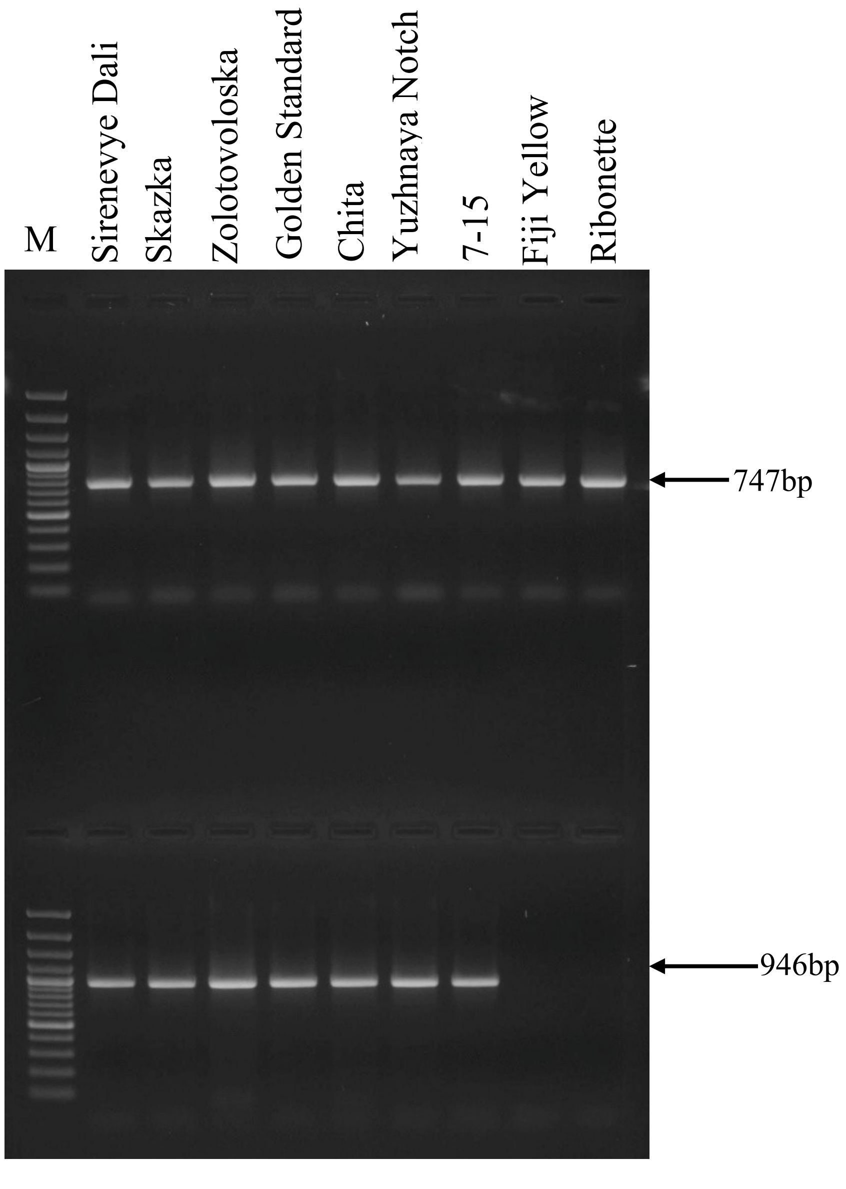
M
Sirenevye Dali
Skazka
Zolotovoloska
Golden Standard
Chita
Yuzhnaya Notch
7-15
Fiji Yellow
Ribonette
747bp
946bp

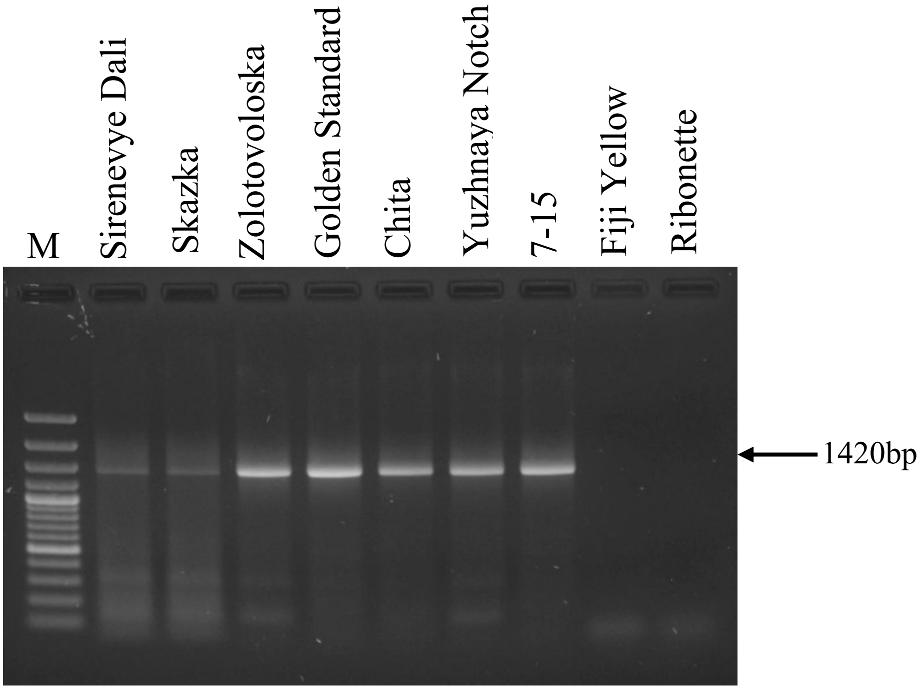

Figure S3. Representaive analysis of RT-PCR products from chrysanthemum virus R (CVR)-infected plants by agarose gel electrophoresis. The names of chrysanthemum cultivars are indicated above the picture. M - GeneRuler 100 bp DNA ladder Plus (Thermo Scientific). The arrows to the right of the picture indicates the CVR-specific PCR products of 747, 946, and 1420 base pairs.
