# Supplementary material for: Highly divergent isolates of chrysanthemum virus B and chrysanthemum virus R infecting chrysanthemum in Russia
Source: PeerJ. 2022 Jan 5;10:e12607. doi: 10.7717/peerj.12607 (PMC8742542; doi:10.7717/peerj.12607)
Supplement: Supplemental Information 5 — A–5’-terminal region of the replicase gene; B–hypervariable region of the replicase gene; C–triple gene block 3 protein gene, intergenic region 2, and coat protein gene. The trees were reconstructed using the neighbour-joining algorithm and Kimura 2-parametric model implemented in MEGA7 (Kumar, Stecher & Tamura, 2016). Names of isolates and their GenBank accession numbers are shown at the end of branches. SK, YN, ZV, SD, Chita, X21, X6, and X13 are isolates from the cultivars Skazka, Yuzhnaya Notch, Zolotovoloska, Sirenevye Dali, Chita, Golden Standard, Ribonette, and Fiji Yellow, respectively. BJ is the Chinese isolate BJ and 7–15 is the Russian isolate from hybrid 7–15. Bootstrap values (>75%) from 1,000 replicates are displayed next to the corresponding nodes. The scale bars indicate the number of substitutions per nucleotide. [file peerj-10-12607-s005.pdf]

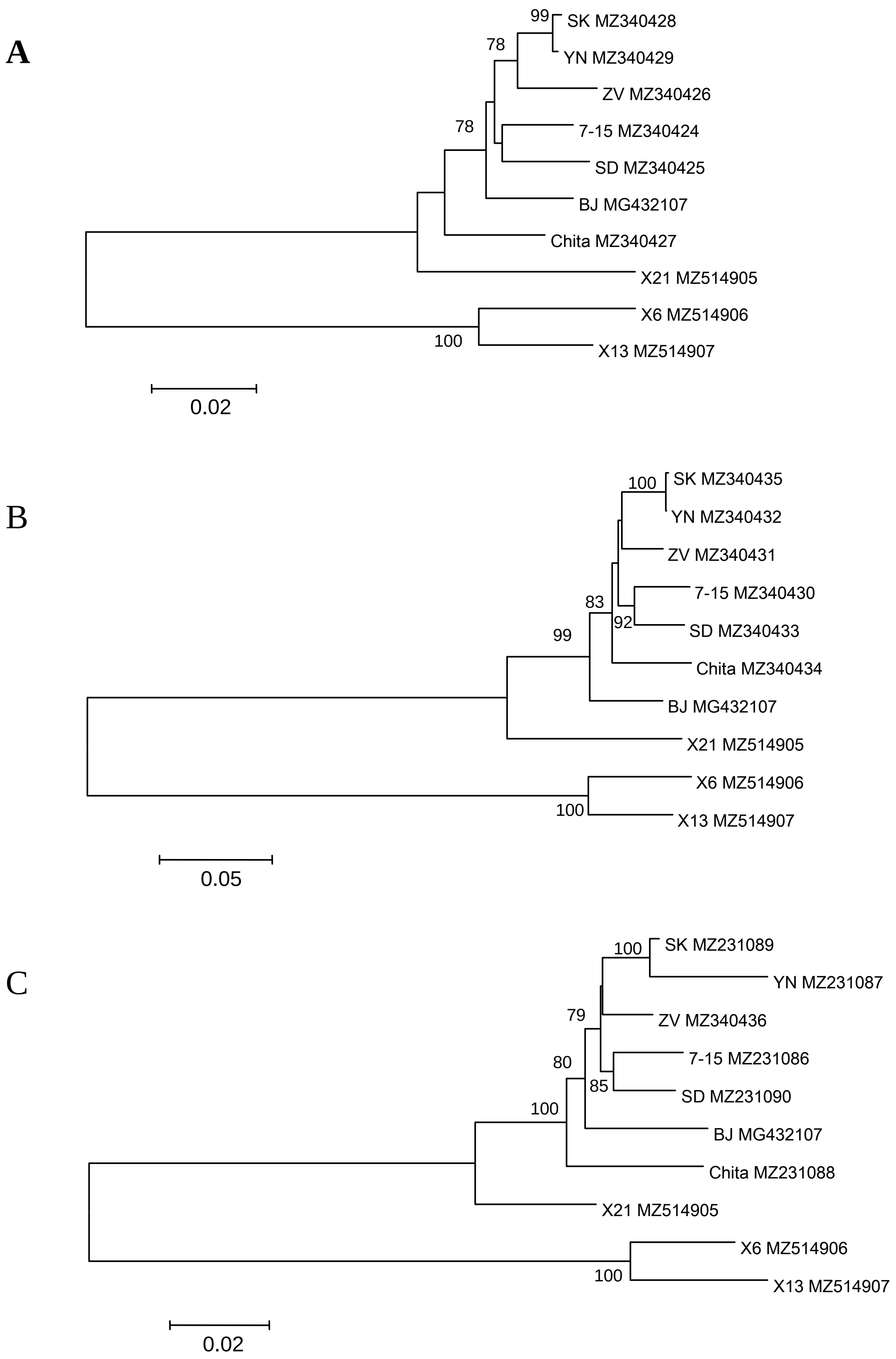

Figure S3. Phylogenetic analysis of three genomic regions of chrysanthemum virus R (CVR) isolates. A – 5'-terminal region of the replicase gene; B – hypervariable region of the replicase gene; C – triple gene block 3 protein, intergenic region 2, and coat protein gene. The

trees were reconstructed using neighbour-joining algorithm and Kimura 2-parametric model implemented in MEGA7 (*Kumar et al., 2016)*. Names of isolates and their GenBank accession numbers are shown at the end of branches. SK, YN, ZV, SD, Chita, X21, X6, and X13 are the CVR isolates from the cultivars Skazka, Yuzhnaya Notch, Zolotovoloska, Sirenevye Dali, Chita, Golden Standard, Ribonette, and Fiji Yellow, respectively. BJ is the Chinese isolate BJ and 7-15 is the Russian isolate from hybrid 7-15. Bootstrap values (>75%) from 1000 replicates are displayed next to the corresponding nodes. The scale bar indicates the number of substitutions per nucleotide.
