# Supplementary material for: Highly divergent isolates of chrysanthemum virus B and chrysanthemum virus R infecting chrysanthemum in Russia
Source: PeerJ. 2022 Jan 5;10:e12607. doi: 10.7717/peerj.12607 (PMC8742542; doi:10.7717/peerj.12607)
Supplement: Supplemental Information 6 — Alignment was employed using ClustalW implemented in MEGA7 program. Names of isolates are presented in the left column. Numerals above the alignment indicate amino acid positions (numbering according to the CVR Chinese isolate BJ replicase, MG432107). Deletions are in blue. C-terminal fragment of 2OG-Fe(II) Oxy domain and N-terminal fragment of helicase domain are in yellow and green, respectively. [file peerj-10-12607-s006.pdf]

```
                                                                642▼   ▼643
BJ              YRTFTQPWKTVVHEVVCVKHTLKKTGNYSWLRCARVAQKHGG--TNEEPIRADRSTETAG  658
X21             YRTFAQPWKFVVHEVVCAKHTLKKTDNYSWLRCAKVVQRHGG--TNGEPTRADGSTETAG  658
X6              YKSFSRPWGVVVHEIVSNKNVLKKTDNYSWLRCKKTIQEEVRDEKYEEKVEPEHDPETGA  660
X13             YKSFSRPWGAVVHEIVSKKIVLKKTDNYSWLRCEKSVKEKVRDIKNETKVETEHGPETGA  660

                           672▼▼673
BJ              GGSQFNEARTDQDNKTNGGDAPNEGEWTPPQGSKYEEMPPPPQRQGSQREEQRAIKCTCN  718
X21             VGAQFNRAKTDQDNKTSGENAHNQGERKSPQDDKYEEMPPPPQWQGRDREDLRTIKCTCD  718
X6              ASTQTEGPQGKKD--SGKEDQPTRDERKNPSEAEHGGTMPPPWCDNNQQEEKHVVKCACE  718
X13             ASTQTEGPQVKKD--SGKEEQPARDERKNPSEAEHGGAMPPPWSDNNQKEEKRVVKCACD  718
```

```
                                                                          894▼ ▼895
BJ              RSIVGPGDLFEMPGGFQETHKHGVCETEKGRLSVTFRVMKKNPHSGEEKDHGASEG-HQN  897
X21             RSKVGPGDLFEMPGGFQETHKHGVCETEKGRLSVTFRVMKKNPHNNEEKDHGASMN-HQE  897
X6              KFKVGPGDLFEMPEGFQITHKHGVRSTSKNRLSVTFRVMKDDHENKKENPTESNADTYDE  898
X13             SFKVGPGDLFEMPEGFQMTHKHGVRSTSKNRLSVTFRVMKDDNKNKKGNSTESNANTHDE  898

                                  921▼▼922
BJ              GTENVNDQSESETHEEKPQQEGVKERESVQESGEGISEEHKQEQSDCPNNGTAGAGTEAF  957
X21             ETEDVNDQSEPETHEEKPQQEGANEGGNVQENCEGVSESHEQEKSYHPDNGAAGVGTEAF  957
X6              GSAGGNGKPEPDGSVPEEPQEGN--GGSEHGSAGSASGSQGQGNDGQNQNHSGKKGTEAF  956
X13             GGAGGNEEPESDGCAPEESQEGS--GGREQGSAGSTSGSQGQGKDGQNQDHSGKKGTEAF  956
```

```
                                                                       1310▼
BJ              VLAVIGTFGSGKSTILKNFFKINLGRKVLYVSPRRALLNEFQRSACGDFKDKKERKNARK  1317
X21             VLAVIGTFGSGKSTILKNFFKINLGRKVLYVSPRRALLNEFQKSACGDFKNKNERKNARK  1317
X6              VLAVIGTFGSGKSTILKNFFKINLGRKVLYVSPRRALLEEFQRGVCGELKAQ-ERRSVRK  1315
X13             VLAVIGTFGSGKSTILKNFFKINLGRKVLYVSPRRALLEEFQRGVCGELKTQ-ERRCVRK  1315
```

Figure S4. Alignment of three regions of the chrysanthemum virus R (CVR) replicase protein. Alignment was employed using ClustalW implemented in MEGA7 program. Names of isolates are presented in the left column. Numerals above the alignment indicate amino acid positions (numbering according to the CVR Chinese isolate BJ replicase, MG432107). Deletions are in blue. C-terminal fragment of 2OG-Fe(II) Oxy domain and N-terminal fragment of helicase domain are in yellow and green, respectively.
