# Supplementary material for: Highly divergent isolates of chrysanthemum virus B and chrysanthemum virus R infecting chrysanthemum in Russia
Source: PeerJ. 2022 Jan 5;10:e12607. doi: 10.7717/peerj.12607 (PMC8742542; doi:10.7717/peerj.12607)
Supplement: Supplemental Information 7 — The Russian isolates are in yellow. The trees were reconstructed using the neighbour-joining algorithm implemented in MEGA7 (Kumar, Stecher & Tamura, 2016). Names of isolates and their GenBank accession numbers are shown at the end of branches. Bootstrap values from 1000 replicates are displayed next to the nodes. The scale bars indicate the number of substitutions per nucleotide. [file peerj-10-12607-s007.pdf]

A

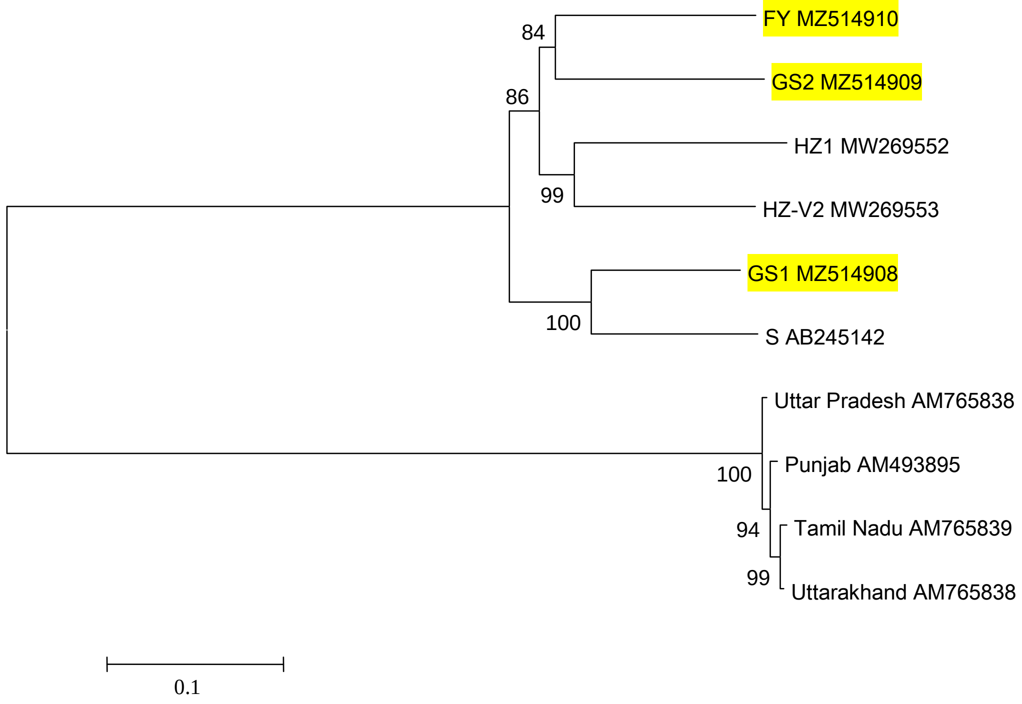

B

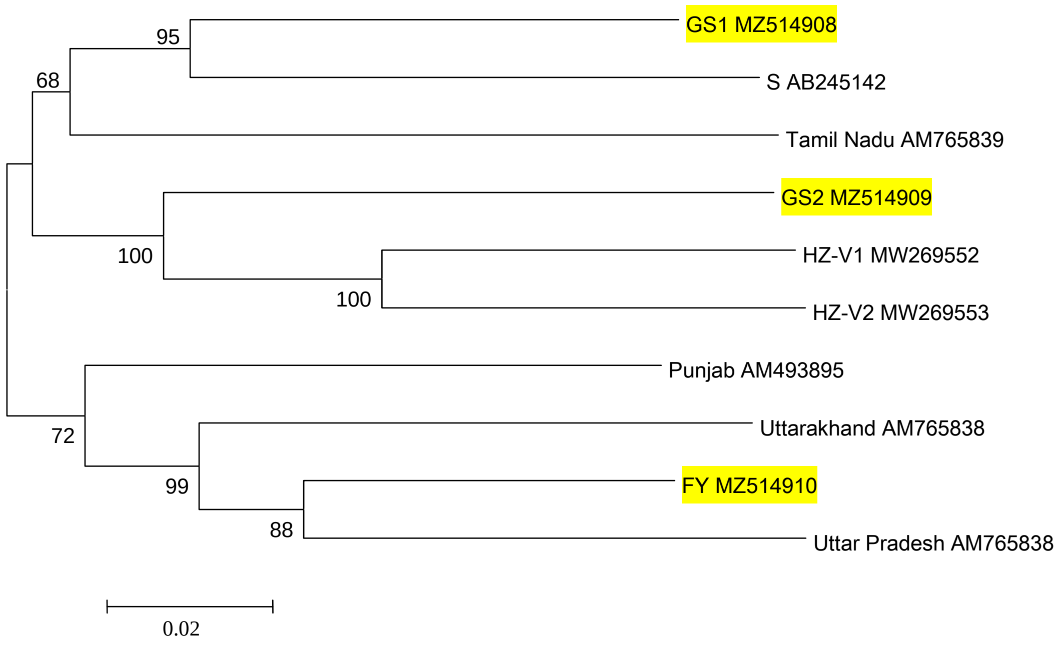

Figure S5. Phylogenetic analysis of chrysanthemum virus B genome regions corresponding to the putative recombinant sequence of the isolate FY (**A**) and to the rest of the genome (**B**). The Russian isolates are in yellow. The trees were reconstructed using the neighbour-joining algorithm implemented in MEGA7 (*Kumar et al., 2016*). Names of isolates and their GenBank accession numbers are shown at the end of branches. Bootstrap values from 1000 replicates are displayed next to the nodes. The scale bars indicate the number of substitutions per nucleotide.
